# IL-37 and Neuroimmune Mechanisms Relevant to Depressive and Anxiety Disorders: A Scoping Review

**DOI:** 10.3390/ijms27146496

**Published:** 2026-07-22

**Authors:** Justyna Kunikowska, Piotr Gałecki, Kuan Pin Su, Katarzyna Bliźniewska-Kowalska, Małgorzata Gałecka

**Affiliations:** 1 Department of Adult Psychiatry, Medical University of Lodz, 91-229 Lodz, Poland; justyna.kunikowska@umed.lodz.pl (J.K.); piotr.galecki@umed.lodz.pl (P.G.); katarzyna.blizniewska-kowalska@umed.lodz.pl (K.B.-K.); 2 College of Medicine, China Medical University (CMU), Taichung 404333, Taiwan; cobol@cmu.edu.tw; 3 Mind-Body Interface Research Center (MBI Lab & Care), CMU Hospital, Taichung 40447, Taiwan; 4 Office of Research and Development, Asia University, Taichung 413305, Taiwan; 5 An-Nan Hospital, China Medical University (CMU), Tainan 709204, Taiwan; 6 Department of Psychotherapy, Medical University of Lodz, 91-229 Lodz, Poland

**Keywords:** interleukin-37, depression, anxiety disorders, neuroinflammation, neuroimmune signaling, cytokines, scoping review

## Abstract

Neuroimmune dysregulation and altered pro- and anti-inflammatory signaling have been implicated in selected phenotypes of depressive and anxiety disorders. Interleukin-37 (IL-37), a member of the IL-1 family, exerts anti-inflammatory effects through extracellular signaling involving IL-18Rα and IL-1R8 and intracellular interactions with SMAD3. This scoping review mapped direct and indirect evidence concerning the relevance of IL-37 to depressive and anxiety disorders. PubMed/MEDLINE was searched through 12 July 2026, supplemented by backward citation searching and reference-list checking; evidence was charted by study type, population or model, IL-37 assessment, principal findings, and level of psychiatric relevance. Thirty-two IL-37-related sources were included. Direct psychiatric evidence comprised one small cross-sectional human study and two rodent stress-model studies, in which IL-37 was assessed as one component of broader inflammatory profiles rather than as a prespecified primary biomarker or intervention target. The remaining evidence was derived from non-psychiatric inflammatory conditions, central nervous system disease models, or mechanistic studies. These findings provide hypothesis-generating biological context but do not establish psychiatric specificity, causality, biomarker validity, or therapeutic efficacy. IL-37 should therefore be considered an exploratory research variable requiring validation in well-characterized longitudinal psychiatric cohorts using standardized assays and integrated immune profiling.

## 1. Introduction

Accumulating evidence suggests that dysregulated communication between the immune system and the central nervous system may contribute to the pathophysiology of selected depressive and anxiety phenotypes [1,2,3]. Proposed mechanisms include chronic low-grade inflammatory activation, alterations in the balance between pro- and anti-inflammatory mediators, microglial responses, oxidative stress, and dysregulation of the hypothalamic–pituitary–adrenal (HPA) axis [2,3]. In some patient subgroups, increased circulating concentrations of pro-inflammatory mediators, including IL-1β, IL-6, and TNF-α, have been reported; however, findings vary across cohorts and do not define a uniform inflammatory phenotype [1,2,3]. Altered peripheral cytokine responses to acute psychosocial stress have also been documented in patients with major depressive disorder [4]. These inflammatory processes may influence neurotransmission, neuroplasticity, cognitive function, and stress-response systems, although the observed associations do not by themselves establish causality.

The relationship between inflammation and depressive and anxiety symptoms is complex, potentially bidirectional, and heterogeneous. Chronic stress may promote immune activation, whereas persistent inflammatory signaling may affect neurobiological systems involved in mood, anxiety, motivation, sleep, and stress processing [2,3]. Available studies further indicate that cytokine profiles may vary according to clinical phenotype. Liang et al. compared cytokine profiles in first-episode drug-naïve patients with major depressive disorder with and without anxiety symptoms [5], whereas Quagliato and Nardi described alterations in pro- and anti-inflammatory mediators in drug-naïve patients with panic disorder [6]. This heterogeneity has prompted interest not only in pro-inflammatory mediators but also in endogenous anti-inflammatory pathways that may counter-regulate excessive immune activation and contribute to neuroimmune homeostasis.

IL-37 is one such mediator. It is a member of the IL-1 family with broad anti-inflammatory and immunoregulatory properties described across inflammatory, autoimmune, and immune-regulatory contexts [7,8,9,10]. From a psychiatric perspective, the central question is not whether IL-37 has general anti-inflammatory activity but whether its expression or function is specifically associated with neuroimmune processes occurring in depressive or anxiety disorders.

Evidence concerning IL-37 in central nervous system diseases suggests a possible role in the regulation of neuroinflammation, but remains limited and indirect [11,12]. Evidence directly linking IL-37 to psychiatric disorders is even more restricted. The current literature comprises one small cross-sectional human study involving patients with major depressive disorder and schizophrenia [13] and two rodent stress-model studies in which IL-37 was assessed within broader inflammatory profiles [14,15]. These sources do not establish psychiatric specificity, causality, biomarker validity, or therapeutic efficacy. IL-37 should therefore be treated as a hypothesis-generating research variable rather than as a validated biomarker or established therapeutic target in depressive or anxiety disorders.

This scoping review aimed to map the extent, characteristics, and evidentiary level of the literature concerning IL-37 biology and neuroimmune mechanisms potentially relevant to depressive and anxiety disorders. It distinguishes direct human psychiatric evidence, stress-related preclinical evidence, and indirect mechanistic evidence derived from non-psychiatric contexts, while identifying major knowledge gaps and methodological limitations. The review is intended to characterize the available evidence base and the corresponding research gap; it does not infer that IL-37 is a validated biomarker or an established therapeutic target in psychiatry.

## 2. Materials and Methods

This study was designed as a scoping review to map and organize the available literature on IL-37 and neuroimmune mechanisms potentially relevant to depressive and anxiety disorders. The scope comprised direct human psychiatric evidence, stress-related preclinical evidence, and indirect mechanistic evidence derived from non-psychiatric inflammatory, autoimmune, and central nervous system contexts. The review was intended to characterize the extent and nature of the available evidence rather than establish IL-37 as a validated biomarker or established therapeutic target.

The reporting of this scoping review was guided by the Preferred Reporting Items for Systematic Reviews and Meta-Analyses extension for Scoping Reviews (PRISMA-ScR) [16]. The identification, screening, full-text eligibility assessment, and inclusion of sources of evidence were presented using the PRISMA 2020 flow diagram [17]. The methodological references describing PRISMA-ScR and PRISMA 2020 were used solely to define the reporting framework and were not included in the substantive synthesis of IL-37-related evidence.

The principal review question was: What is the extent and nature of the direct human psychiatric, stress-related preclinical, and indirect mechanistic evidence concerning IL-37 and neuroimmune processes potentially relevant to depressive and anxiety disorders?

Given the limited number of studies directly assessing IL-37 in patients with clinically diagnosed depressive or anxiety disorders, the review also considered preclinical studies of chronic stress and depressive- or anxiety-like behaviors, together with indirect evidence derived from neuroinflammation, central nervous system diseases, autoimmune conditions, and studies of IL-37 immunoregulatory mechanisms. These sources were included to map the broader biological context of IL-37 and were not interpreted as direct evidence concerning psychiatric patients.

### 2.1. Search Strategy

All searches containing IL-37 terms were conducted in PubMed/MEDLINE on 12 July 2026. The PubMed relative publication-date filter “5 years” was applied together with the Abstract, Full text, Free full text, and language filters (English or Polish). Consequently, the search covered the rolling five-year interval defined by PubMed on the date of retrieval rather than a fixed calendar-date range. Rerunning the same queries at a later date may therefore yield different retrieval counts because the relative date window advances and the content of the database changes over time. The counts reported in this review represent the results obtained on 12 July 2026 and have been retained as the contemporaneous record of the search actually performed. The exact search strings, applied filters, search date, retrieval counts before deduplication, and corresponding export-file identifiers are reported in Supplementary Table S1A.

The search strategy comprised one broad core query containing IL-37 synonyms and nine topic-specific queries addressing depression and anxiety, stress and behavioral models, neuroinflammation and central nervous system diseases, autoimmunity, signaling pathways, lymphocyte responses, oxidative stress, dermatological and allergic diseases, and biomarker-related terms. Within each search block, synonymous or related terms were combined using the Boolean operator OR, whereas the core IL-37 block was combined with each thematic block using AND. The results of all ten searches were exported, merged, and deduplicated before title and abstract screening. Across the ten search blocks, 667 records were retrieved before deduplication. Removal of 392 duplicate records resulted in 275 unique database records for screening.

A separate contextual psychiatric background search was conducted to identify recent publications concerning cytokines, inflammation, neuroinflammation, immune profiles, JAK-STAT signaling, and Th17/Treg-related mechanisms in depressive and anxiety disorders. This contextual search was used only to frame the broader psychiatric and neuroimmune literature and was not included in the PRISMA database record count or screening pool. Contextual records cited in the manuscript were verified separately by PMID and are listed in the Supplementary Materials.

To provide a concise and transparent overview of the database search framework, the ten PubMed/MEDLINE search blocks used for study identification are summarized in Table 1. The table presents the common core terminology used to identify IL-37-related publications and the corresponding thematic terms covering psychiatric, stress-related, neuroinflammatory, immunological, and other biologically relevant contexts. 

The exact PubMed/MEDLINE search strings, applied filters, search dates, and number of records retrieved for each query are provided in Supplementary Table S1A,B. A complete PMID list of all publications included in the descriptive synthesis is provided in Supplementary Table S2.

To assess bibliography completeness, the reference lists of eligible publications were examined. Ten additional IL-37-related reports that were not present in the merged database exports were identified through backward citation searching and reference-list checking. These reports were assessed against the same predefined eligibility criteria as the database-derived records. PubMed searches by title, author, or PMID were used solely to verify bibliographic details and were not treated as an independent method of evidence identification. The ten additional reports and the rationale for their inclusion are reported in Supplementary Table S3A.

A separate contextual psychiatric background search was conducted to identify the recent literature concerning cytokines, inflammation, neuroinflammation, immune profiles, JAK–STAT signaling, and Th17/Treg-related mechanisms in depressive and anxiety disorders. Because this search did not include IL-37 terms, its records were used only to contextualize the broader psychiatric literature and were not treated as sources of evidence, included in the formal screening pool, or counted in the PRISMA flow diagram. The exact search strategy is reported in Supplementary Table S1B, and the eight contextual publications cited in the manuscript are listed in Supplementary Table S3B.

### 2.2. Eligibility Criteria

Eligible sources comprised: clinical or observational studies assessing IL-37 in patients with depressive or anxiety disorders or mixed psychiatric samples containing these diagnostic groups; preclinical studies evaluating IL-37 in chronic stress models or models involving depressive- or anxiety-like behaviors; studies concerning IL-37 in neuroinflammation, microglia, central nervous system diseases, or CNS autoimmunity; and mechanistic studies or reviews addressing IL-37 pathways relevant to the neuroimmune context, including NF-κB/MAPK, SMAD3, IL-18Rα/IL-1R8/SIGIRR, oxidative stress, autophagy, and lymphocyte responses. Contextual psychiatric publications that did not assess IL-37 were used exclusively as background and were not included in the formal set of sources of evidence.

Publications were excluded if IL-37 was not assessed or substantively discussed, if the subject was outside the scope of the review, if only a conference abstract or report without sufficient data was available, or if the full text was unavailable in English or Polish. Records in which the term “37” referred to the number of cytokines analyzed rather than to interleukin-37 were also excluded.

### 2.3. Publication Selection and Data Synthesis

Publication selection was conducted in two stages. First, the titles and abstracts of the identified records were screened. Second, the full texts of potentially eligible publications were assessed against the predefined eligibility criteria. For each included source, the following data were charted: author and year of publication, study type, study population or experimental model, clinical or biological context, method of IL-37 assessment, principal findings concerning IL-37, relevance to depressive or anxiety disorders or related neuroimmune mechanisms, and the main limitations of the study.

All 32 included sources were considered in the descriptive synthesis and are listed in Supplementary Table S2. Focused summaries of the sources most directly relevant to the mechanistic and psychiatric questions of the review are provided later in the manuscript and are not intended to duplicate the complete list presented in Supplementary Table S2.

Given the heterogeneity of the included sources, which comprised clinical studies, preclinical models, mechanistic studies, and reviews, no quantitative synthesis or meta-analysis was performed. The findings were synthesized descriptively and comparatively. Screening and data charting were performed by one author and verified by a second author, with uncertainties resolved through discussion.

### 2.4. PRISMA Flow Diagram

**Figure 1:**
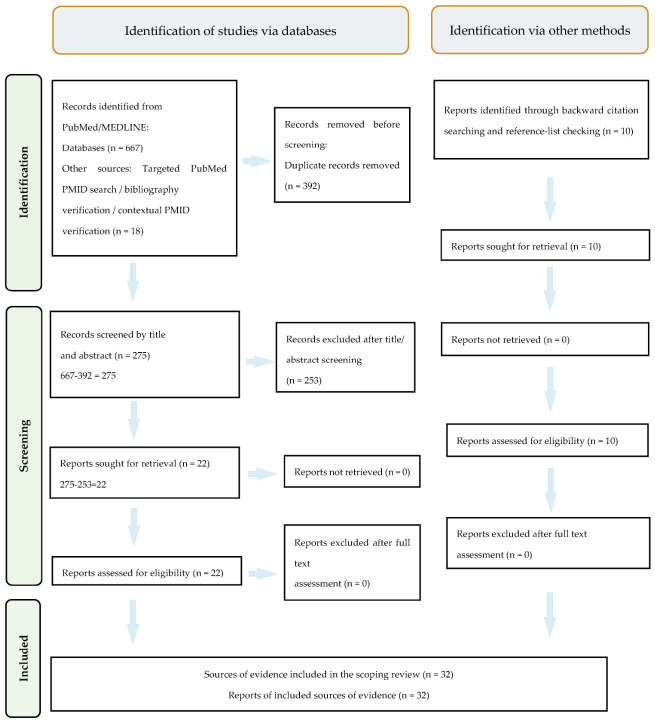
PRISMA 2020 flow diagram illustrating the identification, screening, eligibility assessment, and inclusion of sources of evidence in the scoping review. Database records were identified through ten IL-37-dependent PubMed/MEDLINE search blocks. Additional reports were identified through backward citation searching and reference-list checking; PubMed searches by title, author, and PMID were used solely for bibliographic verification. The separate contextual psychiatric background search and publications that did not assess IL-37 were not included in the PRISMA screening pool. Additional publications identified or verified outside the database screening pool (n = 18). 10 IL-37-related reports included via backward citation searching and 8 contextual psychiatric publications used solely as background and not included in PRISMA.

The study-selection process is presented in Figure 1. The ten IL-37-dependent PubMed/MEDLINE searches yielded 667 records before deduplication. After the removal of 392 duplicate records, 275 unique records were screened by title and abstract; 253 were excluded, leaving 22 reports for retrieval and full-text eligibility assessment. All 22 reports met the predefined inclusion criteria. An additional 10 IL-37-related reports were identified through backward citation searching and reference-list checking; all were retrieved, assessed against the same eligibility criteria, and included. In total, 32 sources of evidence were included in the formal scoping-review synthesis. The separate contextual psychiatric background search and the eight publications that did not assess IL-37 were not included in the PRISMA screening pool or formal evidence set.

In parallel, 10 additional IL-37-related reports that were not present in the database exports were identified through backward citation searching and reference-list checking. PubMed searches by title, author, and PMID were used solely to verify bibliographic details and were not treated as an independent identification method. All 10 additional reports were retrieved and assessed against the same predefined eligibility criteria as the database-derived reports.

No reports were excluded after full-text assessment. In total, 32 sources of evidence were included in the formal scoping-review synthesis. Eight psychiatric publications that did not assess IL-37 were cited solely as contextual background and were not included in the PRISMA screening pool or the formal set of included sources of evidence.

## 3. Cytokine Signaling and Neuroimmune Mechanisms in Depressive and Anxiety Disorders

Complex interactions between the immune system and the central nervous system enable bidirectional communication between these two systems, directly influencing brain function, behavior, and emotional regulation. Cytokines play a particularly important role in this communication axis, as they participate in the regulation of neurobiological processes, including neuroplasticity, neurogenesis, synaptic transmission, and the activity of neuronal circuits involved in mood and stress responsiveness [1,2,3,4]. A growing body of evidence indicates that chronic stress may increase the expression of pro-inflammatory cytokines, highlighting their potential role as a link between stressors, immune activation, and psychiatric symptoms [2,3].

Cytokines may influence brain function through several pathways. First, peripheral inflammatory mediators may affect the central nervous system through regions with increased permeability of the blood–brain barrier, activation of the vagus nerve, and modulation of endothelial and microglial cell activity. Second, cytokines produced locally within the CNS by microglia and astrocytes may directly influence neurotransmitter metabolism, synaptic plasticity, hypothalamic-pituitary-adrenal (HPA) axis activity, and mechanisms of oxidative stress [2,3]. Consequently, chronic inflammatory activation may promote alterations in serotonergic, glutamatergic, and dopaminergic systems, as well as disrupt tryptophan metabolism and the kynurenine pathway, all of which are relevant to the regulation of mood, motivation, anxiety, and cognitive function [2,3].

In depressive and anxiety disorders, particular attention has been paid to pro-inflammatory cytokines such as IL-1β, IL-6, TNF-α, and IFN-γ. Elevated levels of these cytokines may enhance HPA axis activity, affect glucocorticoid signaling, increase microglial activation, and contribute to the persistence of chronic low-grade inflammation. However, available studies indicate substantial heterogeneity in inflammatory responses, depending, among other factors, on symptom phenotype, disease severity, sex, obesity, somatic comorbidities, exposure to chronic stress, and treatment status [1,2,5]. Clinical studies have also shown that cytokine profiles may differ among patients with major depressive disorder depending on the co-occurrence of anxiety symptoms, further emphasizing the need for cautious interpretation of inflammatory markers in psychiatry [5].

The relevance of inflammatory responses has also been described in anxiety disorders. In psychotropic-naïve patients with panic disorder, alterations in cytokine profiles have been demonstrated, including an increase in selected pro-inflammatory mediators and a reduction in the anti-inflammatory cytokine IL-10 [6]. These findings support the concept of disturbed immune balance in certain anxiety phenotypes, although they do not indicate a single shared inflammatory profile across all patients.

Cytokine production and activity are associated with both innate and adaptive immune responses. Contemporary immunological models of depression and anxiety emphasize the importance of the Th17/Treg axis and the balance between pro-inflammatory and anti-inflammatory mediators. Anti-inflammatory cytokines, such as IL-10, IL-4, IL-35, and potentially IL-37, may limit excessive inflammatory activation and contribute to the restoration of immune homeostasis. Studies of major depressive disorder have described alterations in both pro- and anti-inflammatory mediators, including IL-17, IL-21, IL-23, IL-35, and Foxp3, supporting the concept that impaired immune regulation may be involved in certain depressive phenotypes [18]. In addition, the study by Yu et al. indicates an imbalance between pro-inflammatory and anti-inflammatory mediators in patients with MDD, also involving IL-37 [13].

An imbalance between pro-inflammatory and anti-inflammatory mediators may disrupt neuroimmune homeostasis, contributing to cognitive impairment, sleep disturbances, anhedonia, increased stress reactivity, and greater severity of depressive and anxiety symptoms. Studies suggest that the relationship between inflammation and psychiatric symptoms may be partly symptom-specific—that is, stronger for some dimensions of depression and anxiety than for others—which further underscores the need for caution when interpreting inflammatory markers as potential psychiatric biomarkers [1].

In this context, endogenous anti-inflammatory cytokines are of particular importance, as they may serve as compensatory mechanisms in response to chronic immune activation. IL-37, as a cytokine with anti-inflammatory and immunoregulatory properties, fits within this theoretical model. However, its potential relevance in depressive and anxiety disorders should currently be understood primarily as a neuroimmune hypothesis derived from clinical, preclinical, and indirect evidence of translational relevance, rather than as a confirmed pathophysiological mechanism or validated biomarker of these disorders.

## 4. Significance of IL-37 in Other Inflammatory Diseases as Indirect Translational Evidence

Interleukin-37 (IL-37), a member of the IL-1 family, exhibits anti-inflammatory and immunoregulatory properties. Unlike many classical pro-inflammatory cytokines within this family, IL-37 may suppress the production of pro-inflammatory mediators, such as IL-1β, IL-6, and TNF-α, and modulate the activity of immune cells. Current studies emphasize its involvement in the regulation of both innate and adaptive immune responses, as well as its relevance in inflammatory, autoimmune, and neuroinflammatory diseases [7,8,9,10].

**Figure 2:**
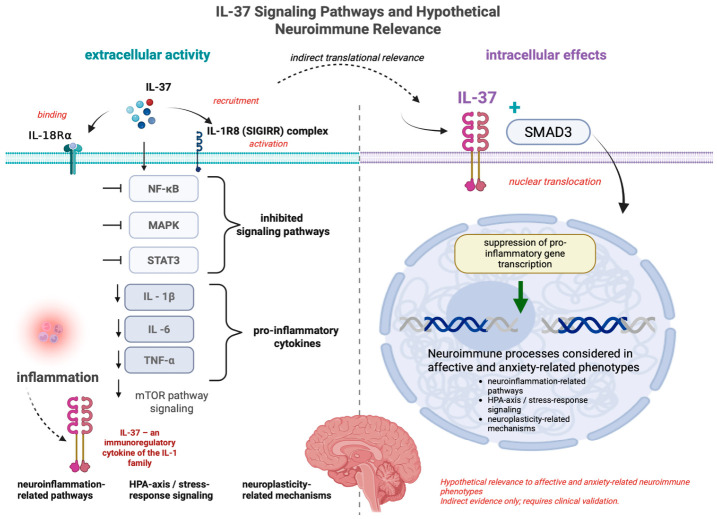
IL-37 signaling pathways and their hypothetical relevance to neuroimmune mechanisms. Solid arrows indicate activation or directional signaling, blunt-ended lines indicate inhibition, and dashed arrows indicate indirect or hypothetical translational relationships. Created with BioRender, (Kunikowska, 2026).

IL-37 exerts its effects through a dual mechanism of action: extracellular and intracellular. Extracellularly, it may bind to IL-18Rα and IL-1R8/SIGIRR, forming a receptor complex associated with the inhibition of inflammatory responses. Intracellularly, IL-37 may interact with SMAD3 and influence the transcription of genes involved in the regulation of inflammatory processes. More recent studies have also highlighted the importance of extracellular and intracellular IL-37 signaling pathways in controlling inflammatory responses, regulating immune cell activity, and maintaining immune homeostasis [7,8,10,19]. Selected IL-37 signaling pathways and their hypothetical relevance to neuroimmune mechanisms are schematically presented in Figure 2.

IL-37 is an IL-1 family cytokine with anti-inflammatory and immunoregulatory properties. Extracellular IL-37 may signal through IL-18Rα and IL-1R8/SIGIRR, forming a receptor complex associated with the attenuation of selected inflammatory pathways, including NF-κB- and MAPK-related signaling. Intracellularly, IL-37 may interact with SMAD3 and influence the transcription of genes involved in inflammatory regulation. These mechanisms have been described mainly in inflammatory, autoimmune, metabolic, vascular, allergic, and experimental neuroinflammatory contexts.

The potential relevance of these pathways to depressive and anxiety disorders remains hypothetical and requires direct clinical validation. This figure should therefore be interpreted as a mechanistic framework for future research, not as evidence that IL-37 reduces depressive or anxiety symptoms, represents a validated biomarker, or constitutes an established therapeutic target in psychiatric disorders.

The relevance of IL-37 has been described in numerous inflammatory, autoimmune, metabolic, cardiovascular, allergic, and neuroinflammatory diseases. In these areas, IL-37 is most commonly analyzed as an endogenous regulator of inflammatory responses, whose expression or activity may vary depending on disease type, inflammatory severity, and the model studied. In autoimmune and inflammatory diseases, IL-37 has primarily been associated with inhibition of pro-inflammatory cytokine production, regulation of NF-κB/MAPK and SMAD3 signaling pathways, and modulation of lymphocyte responses [7,8,20,21,22]. In metabolic and vascular diseases, by contrast, it has been analyzed in the context of chronic low-grade inflammation, oxidative stress, ferroptosis, endothelial dysfunction, and cellular injury [19,23,24].

An important aspect of IL-37 activity is its influence on lymphocyte responses. Experimental studies have demonstrated the involvement of IL-37 in the regulation of the Th1/Th2 and Th17/Treg axes, CD4+ lymphocytes, and regulatory cell populations, including regulatory B cells [22,25,26,27,28,29]. These mechanisms are indirectly relevant to psychiatry, as disturbances in the balance between pro-inflammatory and anti-inflammatory responses, including dysregulation of the Th17/Treg axis, are considered in biological models of certain depressive and anxiety phenotypes.

Of particular relevance to the present review are data concerning IL-37 in central nervous system diseases and neuroinflammatory processes. A review addressing CNS diseases indicated that IL-37 may participate in the regulation of inflammatory responses within the nervous system, although the number of studies in this area remains limited [11]. In a model of Alzheimer’s disease, IL-37 expression reduced acute and chronic neuroinflammation and protected against cognitive decline [12]. In a model of experimental autoimmune encephalomyelitis, IL-37 was shown to attenuate inflammation within the CNS through the IL-1R5/IL-1R8 receptor complex [30]. More recent studies also suggest that IL-37 may inhibit CNS autoimmunity by influencing Treg cells and CD4+ lymphocyte responses, as well as modulate the microglial phenotype and inflammatory response through the MyD88/NF-κB pathway in a model of LPS-induced neuroinflammation [29,31].

Data from the field of neuroinflammation are important from a psychiatric perspective because microglial activation, chronic inflammatory responses within the CNS, cognitive dysfunction, and dysregulation of the neuroimmune axis are considered elements of the pathophysiology of certain depressive and anxiety phenotypes. It should be emphasized, however, that findings from studies on neurodegenerative diseases, CNS autoimmunity, or experimentally induced neuroinflammation cannot be directly extrapolated to psychiatric patient populations. Rather, they represent indirect evidence that may be useful for formulating hypotheses regarding the biological mechanisms of IL-37.

**Figure 3:**
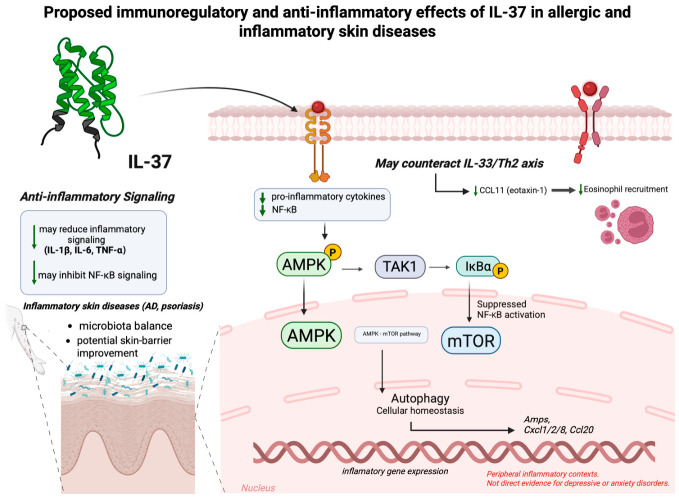
Proposed immunoregulatory and anti-inflammatory effects of IL-37 in allergic and inflammatory skin diseases. Arrows indicate activation or directional effects, downward arrows indicate reduction, and blunt-ended lines indicate inhibition. Created with BioRender, (Kunikowska, 2026).

Data from allergic and dermatological diseases should also be considered, as they clearly illustrate recurring anti-inflammatory mechanisms of IL-37 in peripheral tissues and support the interpretation of Figure 3. In atopic dermatitis, psoriasis, and inflammatory skin diseases, IL-37 has been described as participating in the regulation of Th2/Th17 responses, tissue barrier function, the microbiota, autophagy, and the IL-33–IL-37 axis [32,33,34,35,36,37]. These mechanisms do not constitute direct evidence for the involvement of IL-37 in depression or anxiety disorders, but they demonstrate its recurrent immunoregulatory function in chronic inflammatory conditions. The proposed effects of IL-37 in allergic and inflammatory skin diseases are presented in Figure 3.

IL-37, a member of the IL-1 cytokine family, has been implicated in immunoregulatory and anti-inflammatory processes in peripheral inflammatory tissues. Available evidence suggests that IL-37 may suppress the production of pro-inflammatory cytokines, including IL-1β, IL-6, and TNF-α, and attenuate NF-κB-related signaling. IL-37 has also been associated with modulation of the AMPK–mTOR pathway, potentially contributing to autophagy and cellular homeostasis. In allergic inflammation, IL-37 may counteract the IL-33/Th2 axis, with possible downstream effects on CCL11/eotaxin-1 signaling and eosinophil recruitment. In inflammatory skin diseases, particularly atopic dermatitis and psoriasis, IL-37 has been linked to skin-barrier regulation and microbiota-related mechanisms. These proposed mechanisms illustrate peripheral immunoregulatory functions of IL-37 and should be interpreted as indirect translational context only. They do not constitute direct evidence for the involvement of IL-37 in depressive or anxiety disorders, and their therapeutic relevance requires further experimental and clinical validation.

Data from other inflammatory diseases should be regarded as indirect and interpreted with caution. Their relevance stems from the fact that some mechanisms observed in somatic inflammatory diseases—chronic inflammatory activation, cytokine imbalance, oxidative stress, NF-κB/MAPK activation, alterations in Th17/Treg responses, and neuroinflammatory processes—are also considered in biological models of depression and anxiety disorders. This does not mean, however, that findings from inflammatory diseases can be directly extrapolated to psychiatric populations. Rather, they provide a basis for formulating hypotheses regarding IL-37 as a potential component of the neuroimmune axis, requiring further clinical investigation.

Table 2 presents selected mechanisms of IL-37 activity described in the PubMed literature from 2021–2026 and their possible indirect relevance to research on depressive and anxiety disorders.

## 5. IL-37 in Depressive and Anxiety Disorders: Clinical, Preclinical, and Indirect Neuroimmune Evidence

Clinical studies that did not specifically assess IL-37 have reported heterogeneous cytokine findings in major depressive disorder with and without anxiety symptoms and in panic disorder [5,6].

Liang et al. analyzed a cytokine panel in pharmacologically untreated patients with first-episode major depressive disorder, with and without anxiety symptoms, indicating possible but inconclusive differences in cytokine profiles between these groups [5]. Quagliato and Nardi demonstrated alterations in pro- and anti-inflammatory mediators in patients with panic disorder, including reduced IL-10 levels, supporting the concept of disturbed immune balance in certain anxiety phenotypes [6]. These publications provide contextual psychiatric background for studying endogenous anti-inflammatory mechanisms but do not constitute direct evidence concerning IL-37.

Depressive and anxiety disorders are among the most common mental disorders, and their clinical course is often chronic, recurrent, and heterogeneous. In some patients, somatic symptoms, sleep disturbances, fatigue, cognitive impairment, metabolic disturbances, and features of resistance to standard pharmacological treatment are observed. A growing body of evidence indicates that in selected patient subgroups, these symptoms may be associated with chronic inflammatory activation and an imbalance between pro-inflammatory and anti-inflammatory mediators. Within this broader context, the established anti-inflammatory biology of IL-37 provides a rationale for further investigation, but its involvement in specific depressive or anxiety phenotypes has not been demonstrated.

Despite the biological rationale for this hypothesis, the number of studies directly assessing IL-37 in psychiatric populations remains limited. One of the few clinical studies involving patients with major depressive disorder is the study by Yu et al., in which plasma cytokine profiles and short-chain fatty acids were compared among patients with depression, patients with schizophrenia, and healthy controls [13].

In this study, IL-37 was one of the anti-inflammatory mediators assessed, and its concentration was lower in patients with MDD compared with the control group. This finding may suggest the involvement of insufficient anti-inflammatory mediator activity in certain depressive phenotypes; however, it requires cautious interpretation. The study was cross-sectional, included a limited sample size, and does not allow causal inference or support recognition of IL-37 as a validated biomarker of depression.

With regard to anxiety disorders, data on IL-37 are even more limited. The current literature is dominated by studies on general immune activation, pro-inflammatory cytokines, and neuroinflammation in anxiety, whereas the specific role of IL-37 remains poorly understood. Therefore, the relevance of IL-37 in anxiety disorders should currently be considered primarily on the basis of preclinical evidence and indirect studies concerning stress, neuroinflammation, and regulation of immune responses within the CNS.

Important preclinical data are provided by the study by Chen et al., in which a chronic variable stress model was used in rodents to induce depressive- and anxiety-like behaviors [14]. The authors evaluated the effects of esketamine and fluoxetine on behavior and the plasma profile of inflammatory factors. IL-37 was one of the inflammatory and anti-inflammatory mediators analyzed, and its level changed in the context of chronic stress exposure and the pharmacological interventions applied. These findings indicate that in a rodent model, chronic stress was associated with changes in the profile of inflammatory mediators, including IL-37, in relation to depressive- and anxiety-like symptoms.

Another source of indirect evidence with translational relevance is the study by Zhang L. et al., in which rats fed a high-fat diet and exposed to unpredictable chronic mild stress (HFD + UCMS) were analyzed [15]. This model combined metabolic, inflammatory, and stress-related components. The authors demonstrated that Ginkgo biloba extract affected inflammation in the heart and brain, including through inhibition of the NF-κB pathway and changes in the cytokine profile, including IL-37. The relevance of this study to the present review is indirect: it shows that IL-37 may be part of the anti-inflammatory response in a model combining chronic stress, metabolic disturbances, and inflammatory activation, but it does not constitute direct clinical evidence concerning depression or anxiety in humans.

Data from the field of neuroinflammation also deserve particular attention. In a model of Alzheimer’s disease, IL-37 expression reduced acute and chronic neuroinflammation and protected against cognitive decline [12]. Although this model does not directly concern depression or anxiety disorders, the findings are relevant from a neuroimmune perspective, as they indicate that IL-37 may influence inflammatory processes occurring within the central nervous system. Microglial activation, chronic neuroinflammation, and cognitive dysfunction are also considered in biological models of affective and anxiety disorders.

Additional preclinical data from neuroimmune models further support the hypothesis that IL-37 may participate in the regulation of inflammation within the CNS. In a model of experimental autoimmune encephalomyelitis, IL-37 was shown to attenuate inflammation, neurological deficits, and myelin loss through the IL-1R5/IL-1R8 receptor complex [30]. A more recent study by Yazdani et al. indicates that IL-37 inhibited CNS autoimmunity, among other mechanisms, by increasing the frequency of Treg cells and influencing CD4+ lymphocyte responses [29]. In turn, Zhang J. et al. demonstrated that IL-37 modulates the microglial phenotype and attenuates the inflammatory response in a model of LPS-induced neuroinflammation through the MyD88/NF-κB pathway [31]. These data do not directly address depression or anxiety disorders, but they support the relevance of IL-37 as a potential regulator of neuroinflammatory processes that are considered in pathophysiological models of affective disorders.

Studies concerning the immune profile of patients with major depressive disorder also provide important clinical background for considerations regarding IL-37. Gałecka et al. demonstrated an imbalance between pro-inflammatory and anti-inflammatory mediators in patients with depression, including increased expression of IL-17 and IL-23 and reduced expression of IL-21, IL-35, and Foxp3, suggesting a possible role of the Th17/Treg axis and anti-inflammatory cytokines in the pathophysiology of MDD [18]. In a subsequent study by the same group, components of the JAK-STAT pathway were analyzed in patients with depressive disorders; the authors demonstrated, among other findings, increased JAK3 expression and decreased STAT1 expression compared with controls [41]. Although these studies did not include IL-37 measurements, they support the concept that depression may be associated with disturbances in cytokine regulation and intracellular inflammatory signaling. They therefore provide an important rationale for further investigation of anti-inflammatory cytokines, such as IL-37, in well-characterized populations of patients with depressive disorders.

In summary, direct psychiatric evidence is limited to one small cross-sectional human study, while stress-related preclinical evidence is limited to two animal models in which IL-37 was assessed as one component of broader inflammatory profiles. The remaining evidence is indirect and derives from non-psychiatric CNS and neuroimmune models. Current data do not establish psychiatric specificity, causality, biomarker validity, or therapeutic efficacy; IL-37 should therefore be regarded as an exploratory, hypothesis-generating research variable requiring direct clinical validation.

Table 3 summarizes the direct human psychiatric study, the two stress-related preclinical studies, and selected indirect CNS and neuroimmune models included in this review.

## 6. Limitations of the Current Evidence Base and Review Methodology

The search was restricted to PubMed/MEDLINE, English- or Polish-language publications, and records retrieved using the Abstract, Full text, and Free full text filters. Consequently, relevant publications indexed only in other databases or not captured by these availability filters may have been missed. A methodological limitation of this review is the use of PubMed’s relative “5 years” publication-date filter rather than explicit fixed start and end dates. Because this filter defines a rolling interval relative to the date on which the search is performed, rerunning the same search strategy at a later date may produce different retrieval counts. To support transparency and auditability, the exact search date, complete search strings, applied filters, and original retrieval counts are reported in Supplementary Table S1A. Future reviews and updates of this review should use explicit fixed date boundaries within the search syntax to further improve temporal reproducibility.

Despite growing interest in the role of anti-inflammatory cytokines in depressive and anxiety disorders, current knowledge regarding IL-37 remains limited. The most important limitation is the extremely small direct psychiatric evidence base. It comprises one small cross-sectional human study in which IL-37 was assessed as one component of a broader plasma cytokine profile [13] and two rodent stress-model studies in which IL-37 was similarly evaluated among multiple inflammatory mediators [14,15]. No clinical study directly assessing IL-37 in patients with a diagnosed anxiety disorder was identified. The remaining evidence derives largely from non-psychiatric neuroinflammatory, neurodegenerative, autoimmune, and mechanistic contexts [12,29,30,31].

Therefore, it is currently not possible to determine unequivocally whether changes in IL-37 concentrations represent a cause, a consequence, a compensatory mechanism, or merely an accompanying marker of inflammatory activation.

The lack of longitudinal human studies remains a major limitation. At present, it is unknown whether IL-37 levels change in relation to the severity of depressive or anxiety symptoms, disease phase, remission, relapse, or treatment response. Studies assessing the dynamics of IL-37 following antidepressant or anxiolytic treatment, ketamine/esketamine administration, psychotherapy, or anti-inflammatory interventions are also lacking. Without such data, it is difficult to determine whether IL-37 could serve as a marker of disease state, a marker of biological vulnerability, a component of compensatory responses, or an indicator of treatment response.

Another important issue is the clinical heterogeneity of depression and anxiety disorders. These disorders differ in symptom severity, clinical course, somatic comorbidity, presence of metabolic symptoms, stress exposure, previous treatment, and symptom profile. Current studies indicate that cytokine profiles may vary depending on the presence of anxiety symptoms in patients with major depressive disorder and the type of anxiety disorder [5,6]. Whether associations involving IL-37 differ according to inflammatory phenotype, somatic or metabolic comorbidity, chronic stress exposure, medication status, or treatment resistance remains unknown and should be examined prospectively rather than assumed.

Another limitation concerns the nature of the available preclinical studies. Most data derive from animal models, such as chronic variable stress, metabolic-inflammatory models, LPS-induced neuroinflammation, or models of neurodegenerative diseases and CNS autoimmune disorders [12,13,14,15,29,30,31]. These models provide important information on neuroimmune mechanisms; however, they do not fully reflect the clinical complexity of depression and anxiety disorders in humans. Particular caution is required when extrapolating findings from neuroinflammatory, neurodegenerative, or autoimmune models to affective disorders, as the similarity of certain inflammatory pathways does not imply identity of pathophysiological mechanisms.

An additional barrier is the lack of full standardization in IL-37 measurement. Studies differ in terms of biological material, laboratory methods, timing of sample collection, assay sensitivity, and reporting of results. IL-37 may be assessed in serum, plasma, peripheral blood mononuclear cells, or tissue models, which makes comparisons across studies difficult. Clear reference ranges or cut-off values that would allow IL-37 to be interpreted as a clinical marker have not yet been established. Attempts to determine reference ranges for circulating IL-37 in healthy adults indicate that measurement standardization is a necessary prerequisite for future biomarker studies [42].

The distinction between peripheral and central immune responses should also be considered. Most human studies are based on cytokine measurements in peripheral blood, whereas processes relevant to depression and anxiety may occur within the central nervous system, involving microglia, astrocytes, the blood–brain barrier, and the neuroendocrine axis. IL-37 levels in the blood do not necessarily directly reflect its activity within the CNS. Therefore, the interpretation of peripheral IL-37 concentrations as an indicator of neuroinflammation requires particular caution.

## 7. Conclusions

IL-37 is a cytokine with anti-inflammatory and immunoregulatory properties, whose activity includes, among other mechanisms, suppression of pro-inflammatory cytokine production, modulation of NF-κB/MAPK pathways, interaction with SMAD3, effects on lymphocyte responses, and participation in the regulation of inflammatory processes in autoimmune and neuroinflammatory diseases. These mechanisms overlap with pathways investigated in selected depressive and anxiety phenotypes, including chronic inflammatory activation, oxidative stress, microglial responses, and altered pro- and anti-inflammatory signaling. However, this mechanistic overlap does not establish an IL-37-specific role in psychiatric disorders.

However, the available data directly concerning IL-37 in psychiatry remain limited. The direct psychiatric evidence comprises one small cross-sectional study including patients with major depressive disorder, patients with schizophrenia, and healthy controls, in which IL-37 was assessed as one of several plasma cytokines [13]. Two rodent stress-model studies provide preclinical evidence [14,15], whereas the remaining evidence derives from non-psychiatric models of neuroinflammation, microglial responses, CNS autoimmunity, and central nervous system diseases [12,29,30,31]. No clinical study directly assessing IL-37 in patients with a diagnosed anxiety disorder was identified. Taken together, these studies provide limited and heterogeneous evidence compatible with a possible immunoregulatory role of IL-37 in stress- and neuroinflammation-related contexts. They do not establish an IL-37-specific mechanism in depressive or anxiety disorders.

However, they do not allow a clear determination of whether IL-37 alterations are causal, compensatory, or merely associated with inflammatory activation.

At the current stage, IL-37 should not be regarded as a validated biomarker of depression or anxiety disorders, nor as an established therapeutic target. Its relevance should be understood as a promising but still preliminary area of research on the neuroimmune axis, requiring rigorous clinical validation. Further investigation of IL-37 appears particularly important in well-characterized patient groups, especially among individuals with a pronounced inflammatory component, somatic symptoms, metabolic disturbances, chronic stress exposure, or treatment resistance.

Future studies should include larger clinical populations, longitudinal and interventional designs, standardized methods for IL-37 assessment, and simultaneous evaluation of other pro- and anti-inflammatory mediators. It is important to determine whether IL-37 may serve as a marker of specific immunological phenotypes of depression and anxiety, a marker of treatment response, or merely one component of a broader cytokine profile. Such studies are required to determine whether IL-37 has any reproducible value for pathophysiological research, patient stratification, or treatment-response monitoring in depressive and anxiety disorders.

## Figures and Tables

**Table 1:** Search strategy blocks used in PubMed/MEDLINE. The core IL-37 search terms were combined with thematic terms using the Boolean operator AND, while terms within each block were combined using OR. PubMed filters included 5 years, Abstract, Full text, and Free full text. The final search was conducted on 12 July 2026.

Search Block	Core Search Terms	Thematic Terms Combined with AND
main IL-37 term	“IL-37” OR “interleukin-37” OR “interleukin 37” OR “IL37” OR “IL37 protein, human”	—
depression and anxiety	“IL-37” OR “interleukin-37” OR “interleukin 37” OR “IL37” OR “IL37 protein, human”	depression, major depressive disorder, MDD, anxiety, panic disorder, anxiety-like
stress and behavioral models	“IL-37” OR “interleukin-37” OR “interleukin 37” OR “IL37” OR “IL37 protein, human”	chronic stress, chronic variable stress, depression-like behavior, anxiety-like behavior, unpredictable chronic mild stress
neuroinflammation/CNS	“IL-37” OR “interleukin-37” OR “interleukin 37” OR “IL37” OR “IL37 protein, human”	neuroinflammation, microglia, central nervous system, CNS, EAE, Alzheimer
autoimmunology	“IL-37” OR “interleukin-37” OR “interleukin 37” OR “IL37” OR “IL37 protein, human”	autoimmune diseases, autoimmunity, lupus, SLE, arthritis, rheumatoid
signaling pathways	“IL-37” OR “interleukin-37” OR “interleukin 37” OR “IL37” OR “IL37 protein, human”	NF-κB, NF-kB, MAPK, MyD88, SMAD3, IL-1R8, SIGIRR, IL-18R, signaling
lymphocyte response	“IL-37” OR “interleukin-37” OR “interleukin 37” OR “IL37” OR “IL37 protein, human”	Treg, Th17, Th1, Th2, CD4, Breg, regulatory B cells, lymphocyte, T cell
oxidative stress	“IL-37” OR “interleukin-37” OR “interleukin 37” OR “IL37” OR “IL37 protein, human”	oxidative stress, ferroptosis, apoptosis, endothelial, atherosclerosis, coronary, NRF2
dermatology and allergology	“IL-37” OR “interleukin-37” OR “interleukin 37” OR “IL37” OR “IL37 protein, human”	atopic dermatitis, psoriasis, skin, allergic, allergy, IL-33, Th2, microbiota, autophagy
biomarker	“IL-37” OR “interleukin-37” OR “interleukin 37” OR “IL37” OR “IL37 protein, human”	biomarker, serum, plasma, circulating, reference range

**Table 2:** Biological mechanisms of IL-37 described in PubMed-indexed literature from 2021–2026 and their potential indirect relevance to depressive and anxiety disorders.

Mechanism of IL-37 Activity	Area/Model in Which the Mechanism Has Been Described	Potential Relevance to Depression and Anxiety	Publications Supporting the Mechanism
suppression of pro-inflammatory cytokine production, including IL-1β, IL-6, and TNF-α	inflammatory diseases; autoimmune diseases; immunological cell models; regulation of human immune cells	IL-1β, IL-6, and TNF-α are among the mediators most frequently analyzed in models of neuroinflammation, chronic stress, depression, and anxiety. IL-37 may act as a counter-regulatory factor against excessive inflammatory activation.	Zeng et al., 2022 [8] Su and Tao, 2021 [7] Gu et al., 2023 [38] Teufel et al., 2024 [9] Li Y. et al., 2025 [10]
dual mechanism of action: extracellular signaling through IL-18Rα/IL-1R8/SIGIRR and intracellular signaling through SMAD3	inflammatory, autoimmune, vascular, and metabolic diseases	This mechanism provides a biological rationale for investigating IL-37 as a regulator of inflammatory responses. In psychiatry, its relevance is indirect, as similar pro- and anti-inflammatory signaling pathways are analyzed in neuroimmune models of depression and anxiety.	Su and Tao, 2021 [7] Zeng et al., 2022 [8] Mesjasz et al., 2023 [35] Zhang C. et al., 2024 [19] Li Y. et al., 2025 [10]
inhibition of NF-κB and MAPK pathway activation	models of inflammatory diseases; tissue injury; chronic stress; LPS-induced neuroinflammation; microglial activation	NF-κB and MAPK are involved in the regulation of pro-inflammatory cytokines and stress responses. Their activation is considered in models of neuroinflammation and affective disorders.	Su and Tao, 2021 [7] Fu et al., 2025 [39] Zhang L. et al., 2022 [15] Zhang J. et al., 2025 [31] Li Y. et al., 2025 [10]
reduction of oxidative stress, ferroptosis, apoptosis, and cellular injury	Cardiovascular diseases; endothelial dysfunction; chronic low-grade inflammation; macrophage models; diabetic atherosclerosis	Oxidative stress and cellular injury may contribute to neuroinflammation, impaired neuroplasticity, and affective symptoms. However, these indirect findings cannot be directly extrapolated to psychiatric populations.	Rafiei et al., 2022 [23] Zhang C. et al., 2024 [19] Xu J. et al., 2023 [24]
regulation of lymphocyte responses, including the Th1/Th2 and Th17/Treg axes, CD4+ T-cell activity, and regulatory B cells	endometriosis; transplantation; graft rejection models; CNS autoimmunity; rheumatoid arthritis; human regulatory B cells	Disturbances in the Th17/Treg balance and chronic lymphocyte activation are considered components of immune dysregulation in some depressive and anxiety phenotypes. Data concerning Bregs and Tregs strengthen the biological rationale for studying IL-37 as an anti-inflammatory mediator.	Li L. et al., 2021 [25] Shao et al., 2024 [27] Qin et al., 2024 [28] Yazdani et al., 2024 [29] Lyu S. et al., 2024 [40] Su Z. et al., 2023 [26] Wang L. et al., 2025 [22]
regulation of CNS neuroinflammation, microglial activation, and CNS autoimmunity	central nervous system diseases; Alzheimer’s disease model; CNS autoimmunity	Neuroinflammation and microglial activation are considered in the pathophysiology of depression and anxiety. This is the most relevant indirect area for psychiatry, as microglial activation, neuroinflammation, and cognitive dysfunction are considered in biological models of depression and anxiety disorders.	Sánchez-Fernández A. et al., 2021 [30] Li X. et al., 2022 [11] Lonnemann et al., 2022 [12] Yazdani et al., 2024 [29] Zhang J. et al., 2025 [31]
modulation of autophagy, tissue barrier function, the microbiota, the IL-33-IL-37 axis, Th2/Th17 responses, and dermatological-allergic mechanisms	atopic dermatitis; psoriasis; allergic rhinitis; skin diseases; allergic diseases	These data demonstrate recurrent peripheral immunoregulatory mechanisms of IL-37, which may be relevant to a broader interpretation of its anti-inflammatory functions, particularly in relation to Figure 3.	Zhou J. et al., 2021 [32] Mesjasz et al., 2023 [35] Borgia et al., 2022 [33] Wulamujiang et al., 2023 [36] Tsuji G. et al., 2023 [34] Rusiñol and Puig, 2024 [37]
dysregulation of IL-37 in autoimmune and inflammatory diseases and its possible compensatory function	systemic lupus erythematosus; rheumatoid arthritis; autoimmune diseases	Autoimmune diseases show that IL-37 may change in the course of chronic immune activation. In the context of depression and anxiety, these data are indirect, but they support the hypothesis that IL-37 may represent a marker of disturbed pro-inflammatory/anti-inflammatory balance.	Wang L. et al., 2025 [22] Zeng et al., 2022 [8] Xu Y. et al., 2024 [20] Lyu S. et al., 2024 [40] Lee and Song, 2025 [21]

**Table 3:** Evidence concerning IL-37 in depressive and anxiety disorders and related neuroimmune contexts: clinical, preclinical, and indirect data from 2021–2026.

Author	Type of Evidence	Study Group/Model	Role of IL-37	Relevance to Depression/Anxiety	Limitations
Yu et al., 2024 [13]	Clinical, cross-sectional	Patients with major depressive disorder (MDD), patients with schizophrenia, and a control group	IL-37 concentrations were lower in patients with MDD compared with the control group.	The most direct clinical signal suggesting possible dysregulation of anti-inflammatory mediators, including IL-37, in MDD.	Small study sample. Cross-sectional design. No causal conclusions can be drawn.
Chen et al., 2024 [14]	Preclinical	Mouse model of chronic variable stress, with preliminary assessment of esketamine and fluoxetine	IL-37 was one of the anti-inflammatory mediators analyzed in plasma; its level changed in relation to chronic stress exposure and pharmacological interventions.	A model combining stress, depressive- and anxiety-like features, and immune response. Supports the hypothesis of IL-37 involvement in the stress-inflammation-behavior axis.	Animal model. No human population. No IL-37-targeted therapeutic intervention. Findings cannot be directly extrapolated to psychiatric patients.
Zhang L. et al., 2022 [15]	Preclinical/translational	Rats fed a high-fat diet and exposed to chronic stress. Intervention with *Ginkgo biloba* extract.	IL-37 was assessed as part of the anti-inflammatory profile. The intervention affected inflammatory markers in the brain and heart, including through inhibition of NF-κB.	A metabolic-stress model relevant to the stress–inflammation–CNS axis. May be particularly important for depressive-anxiety phenotypes with a metabolic or inflammatory component.	No psychiatric patient population. No IL-37-specific intervention. Data are indirect.
Lonnemann et al., 2022 [12]	Preclinical, neuroinflammatory	Model of acute and chronic neuroinflammation. Mouse model of Alzheimer’s disease.	IL-37 expression reduced acute and chronic neuroinflammation and protected cognitive function.	Important evidence concerning IL-37 activity in the CNS. Neuroinflammation, microglial activation, and cognitive dysfunction are also considered in biological models of depression and anxiety.	Neurodegenerative model, not a depression or anxiety model. Preclinical data. No direct psychiatric evidence.
Sánchez-Fernández et al., 2021 [30]	Preclinical, neuroimmunology	Experimental autoimmune encephalomyelitis (EAE) model	IL-37 attenuated inflammation, neurological deficits, and myelin loss in the EAE model. Its effect depended on the IL-1R5/IL-1R8 receptor complex.	Indirect evidence indicating that IL-37 may modulate inflammation within the CNS. Relevant to understanding its role in the neuroimmune axis.	CNS autoimmunity model. Not a depression/anxiety model. Limited ability to extrapolate findings to psychiatric populations.
Yazdani et al., 2024 [29]	Preclinical, neuroimmunology	CNS autoimmunity model	IL-37 inhibited CNS autoimmunity by increasing the frequency of Treg cells and influencing CD4+ lymphocyte responses.	Strengthens the indirect link between IL-37 and regulation of immune responses within the CNS. Treg/CD4+ responses and neuroinflammation are relevant to broader neuroimmune models.	No direct assessment of depressive or anxiety symptoms. Autoimmune rather than psychiatric model.
Zhang J. et al., 2025 [31]	Preclinical, mechanistic	LPS-induced neuroinflammation; microglia	IL-37 modulated the microglial phenotype and inhibited the inflammatory response through the MyD88/NF-κB pathway.	Important evidence concerning microglia and neuroinflammation, which are mechanisms considered in the pathophysiology of depressive and anxiety disorders.	LPS-induced inflammation model. No psychiatric population.

## Data Availability

No new data were created or analyzed in this study. Data sharing is not applicable to this article.

## Supplementary Materials

The following supporting information can be downloaded at: https://www.mdpi.com/article/10.3390/ijms27146496/s1.
