# Supplementary material for: IL-37 and Neuroimmune Mechanisms Relevant to Depressive and Anxiety Disorders: A Scoping Review
Source: Int J Mol Sci. 2026 Jul 22;27(14):6496. doi: 10.3390/ijms27146496 (PMC13409887; doi:10.3390/ijms27146496)
Supplement: Supplementary file 1 [file ijms-27-06496-s001.zip › Supplementary_Table_S1A.pdf]

Supplementary Table S1A. PubMed/MEDLINE IL-37-dependent search strategy included in the PRISMA screening pool.

All searches were conducted in PubMed/MEDLINE. The table presents the exact IL-37-dependent search strings used for each thematic search block, the filters applied, the search date, and the number of records retrieved before deduplication.
The results of these searches were exported, merged, and deduplicated before title and abstract screening. The contextual psychiatric background search did not include IL-37 terms and was not included in the PRISMA screening pool; it is reported separately in Supplementary Table S1B.

Search ID	Search block	Exact PubMed/MEDLINE query	Filters applied	Search date	Records retrieved before deduplication	Export file
S1A.1	Main IL-37 term	("IL-37"[Title/Abstract] OR "interleukin-37"[Title/Abstract] OR "interleukin 37"[Title/Abstract] OR "IL37"[Title/Abstract] OR "IL37 protein, human"[Supplementary Concept])	PubMed filters:<br>5 years; Abstract; Full text; Free full text; language: English or Polish	12 July 2026	275	S_1.txt<br>S_1.csv
S1A.2	Depression and anxiety	("IL-37"[Title/Abstract] OR "interleukin-37"[Title/Abstract] OR "interleukin 37"[Title/Abstract] OR "IL37"[Title/Abstract] OR "IL37 protein, human"[Supplementary Concept]) AND ("depression"[Title/Abstract] OR "major depressive disorder"[Title/Abstract] OR	PubMed filters: 5 years; Abstract; Full text; Free full text; language: English or Polish	12 July 2026	3	S_2.txt<br>S_2.csv

		"MDD"[Title/Abstract] OR "anxiety"[Title/Abstract] OR "panic disorder"[Title/Abstract] OR "anxiety-like"[Title/Abstract])				
S1A.3	Stress and behavioral models	("IL-37"[Title/Abstract] OR "interleukin-37"[Title/Abstract] OR "interleukin 37"[Title/Abstract] OR "IL37"[Title/Abstract] OR "IL37 protein, human"[Supplementary Concept]) AND ("chronic stress"[Title/Abstract] OR "chronic variable stress"[Title/Abstract] OR "depression-like behavior"[Title/Abstract] OR "anxiety-like behavior"[Title/Abstract] OR "unpredictable chronic mild stress"[Title/Abstract])	PubMed filters: 5 years; Abstract; Full text; Free full text; language: English or Polish	12 July 2026	2	S_3.txt S_3.csv
S1A.4	Neuroinflammation / CNS	("IL-37"[Title/Abstract] OR "interleukin-37"[Title/Abstract] OR "interleukin 37"[Title/Abstract] OR "IL37"[Title/Abstract] OR "IL37 protein, human"[Supplementary Concept]) AND ("neuroinflammation"[Title/Abstract] OR "microglia"[Title/Abstract] OR "central nervous system"[Title/Abstract] OR	PubMed filters: 5 years; Abstract; Full text; Free full text; language: English or Polish	12 July 2026	8	S_4.txt S_4.csv

		"CNS"[Title/Abstract] OR "EAE"[Title/Abstract] OR "Alzheimer"[Title/Abstract])				
S1A.5	Autoimmunology	("IL-37"[Title/Abstract] OR "interleukin-37"[Title/Abstract] OR "interleukin 37"[Title/Abstract] OR "IL37"[Title/Abstract] OR "IL37 protein, human"[Supplementary Concept]) AND ("autoimmune diseases"[Title/Abstract] OR "autoimmunity"[Title/Abstract] OR "lupus"[Title/Abstract] OR "SLE"[Title/Abstract] OR "arthritis"[Title/Abstract] OR "rheumatoid"[Title/Abstract])	PubMed filters: 5 years; Abstract; Full text; Free full text; language: English or Polish	12 July 2026	32	S_5.txt S_5.csv
S1A.6	Signaling pathways	("IL-37"[Title/Abstract] OR "interleukin-37"[Title/Abstract] OR "interleukin 37"[Title/Abstract] OR "IL37"[Title/Abstract] OR "IL37 protein, human"[Supplementary Concept]) AND ("NF-κB"[Title/Abstract] OR "NF-kB"[Title/Abstract] OR "MAPK"[Title/Abstract] OR "MyD88"[Title/Abstract] OR "SMAD3"[Title/Abstract] OR "IL-1R8"[Title/Abstract] OR "SIGIRR"[Title/Abstract] OR "IL-	PubMed filters: 5 years; Abstract; Full text; Free full text; language: English or Polish	12 July 2026	79	S_6.txt S_6.csv

		18R"[Title/Abstract] OR "signaling"[Title/Abstract])				
S1A.7	Lymphocyte response	("IL-37"[Title/Abstract] OR "interleukin-37"[Title/Abstract] OR "interleukin 37"[Title/Abstract] OR "IL37"[Title/Abstract] OR "IL37 protein, human"[Supplementary Concept]) AND ("Treg"[Title/Abstract] OR "Th17"[Title/Abstract] OR "Th1"[Title/Abstract] OR "Th2"[Title/Abstract] OR "CD4"[Title/Abstract] OR "Breg"[Title/Abstract] OR "regulatory B cells"[Title/Abstract] OR "lymphocyte"[Title/Abstract] OR "T cell"[Title/Abstract])	PubMed filters: 5 years; Abstract; Full text; Free full text; language: English or Polish	12 July 2026	44	S_7.txt S_7.csv
S1A.8	Oxidative stress	("IL-37"[Title/Abstract] OR "interleukin-37"[Title/Abstract] OR "interleukin 37"[Title/Abstract] OR "IL37"[Title/Abstract] OR "IL37 protein, human"[Supplementary Concept]) AND ("oxidative stress"[Title/Abstract] OR "ferroptosis"[Title/Abstract] OR "apoptosis"[Title/Abstract] OR "endothelial"[Title/Abstract] OR "atherosclerosis"[Title/Abstract] OR	PubMed filters: 5 years; Abstract; Full text; Free full text; language: English or Polish	12 July 2026	46	S_8.txt S_8.csv

		"coronary"[Title/Abstract] OR "NRF2"[Title/Abstract])				
S1A.9	Dermatology and allergology	("IL-37"[Title/Abstract] OR "interleukin-37"[Title/Abstract] OR "interleukin 37"[Title/Abstract] OR "IL37"[Title/Abstract] OR "IL37 protein, human"[Supplementary Concept]) AND ("atopic dermatitis"[Title/Abstract] OR "psoriasis"[Title/Abstract] OR "skin"[Title/Abstract] OR "allergic"[Title/Abstract] OR "allergy"[Title/Abstract] OR "IL-33"[Title/Abstract] OR "Th2"[Title/Abstract] OR "microbiota"[Title/Abstract] OR "autophagy"[Title/Abstract])	PubMed filters: 5 years; Abstract; Full text; Free full text; language: English or Polish	12 July 2026	77	S_9.txt S_9.csv
S1A.10	Biomarker	("IL-37"[Title/Abstract] OR "interleukin-37"[Title/Abstract] OR "interleukin 37"[Title/Abstract] OR "IL37"[Title/Abstract] OR "IL37 protein, human"[Supplementary Concept]) AND ("biomarker"[Title/Abstract] OR "serum"[Title/Abstract] OR "plasma"[Title/Abstract] OR "circulating"[Title/Abstract] OR "reference range"[Title/Abstract])	PubMed filters: 5 years; Abstract; Full text; Free full text; language: English or Polish	12 July 2026	101	S_10.txt S_10.csv

Across the ten IL-37-dependent search blocks, 667 records were retrieved before deduplication. After the exports had been merged, 392 duplicate records were removed, leaving 275 unique database records for title and abstract screening.
