# Supplementary material for: IL-37 and Neuroimmune Mechanisms Relevant to Depressive and Anxiety Disorders: A Scoping Review
Source: Int J Mol Sci. 2026 Jul 22;27(14):6496. doi: 10.3390/ijms27146496 (PMC13409887; doi:10.3390/ijms27146496)
Supplement: Supplementary file 1 [file ijms-27-06496-s001.zip › Supplementary_Table_S1B.pdf]

Supplementary Table S1B.

Contextual psychiatric background search not included in the PRISMA screening pool.

A separate contextual psychiatric background search was conducted to identify recent literature concerning cytokine profiles, inflammatory cytokines, immune profiles, JAK-STAT signaling, and Th17/Treg-related mechanisms in depressive and anxiety disorders. Because this search did not include IL-37 terms and was used only to frame the broader psychiatric and neuroimmune literature, it was not included in the PRISMA database-record count or screening pool. Contextual publications cited in the manuscript were verified separately by PMID.

Search ID	Search block	Exact PubMed/MEDLINE query	Filters applied	Search date	Records retrieved	Included in PRISMA screening pool	Purpose
S1B.1	Contextual psychiatric background	("major depressive disorder"[Title/Abstract] OR "depression"[Title/Abstract] OR "panic disorder"[Title/Abstract] OR "anxiety disorder"[Title/Abstract]) AND ("cytokine profile"[Title/Abstract] OR "inflammatory cytokines"[Title/Abst	Publication date: 2021 - 2026; language: English or Polish; Abstract; Full text; Free full text	12 June 2026	n = 868	No	Contextual background only; used to frame the broader psychiatric and neuroimmune literature concerning cytokine profiles, inflammatory signaling, and immune dysregulation in

		ract] OR "immune profile"[Title/Abstract] OR "JAK-STAT"[Title/Abstract] OR "Th17/Treg"[Title/Abstract] OR "Th17"[Title/Abstract] OR "Treg"[Title/Abstract])					depressive and anxiety disorders.

This contextual search did not include IL-37 terms and was used solely to frame the broader psychiatric and neuroimmune literature. Its 868 records were not added to the PRISMA database-record count, were not screened as part of the formal review pool, and were not included in the final count of sources of evidence.
