# Supplementary material for: IL-37 and Neuroimmune Mechanisms Relevant to Depressive and Anxiety Disorders: A Scoping Review
Source: Int J Mol Sci. 2026 Jul 22;27(14):6496. doi: 10.3390/ijms27146496 (PMC13409887; doi:10.3390/ijms27146496)
Supplement: Supplementary file 1 [file ijms-27-06496-s001.zip › Supplementary_Table_S2.pdf]

Supplementary Table S2. PMID list of sources of evidence included in the formal scoping-review synthesis (n = 32).

No.	Reference no. in manuscript	PMID	First author	Year	Identification source	Evidence category
1	[1]	35837057	Zeng	2022	PubMed/MEDLINE database search	IL-37 biology / autoimmune disease
2	[2]	34248996	Su	2021	Backward citation searching/reference-list checking	IL-37 biology / human health and disease
3	[3]	37928545	Gu	2023	PubMed/MEDLINE database search	IL-37 biology / inflammatory and cancer-related mechanisms
4	[4]	38067193	Mesjasz	2023	PubMed/MEDLINE database search	Dermatology / atopic dermatitis
5	[5]	38359489	Zhang C.	2024	PubMed/MEDLINE database search	IL-37 - SMAD3 / endothelial dysfunction
6	[6]	40691258	Fu	2025	PubMed/MEDLINE database search	MAPK - NF-κB inflammatory signaling
7	[7]	35677735	Zhang L.	2022	PubMed/MEDLINE database search	Chronic stress / inflammation / brain - heart model
8	[8]	39307482	Teufel	2024	PubMed/MEDLINE database search	Human immune cell regulation
9	[9]	35636779	Rafiei	2022	PubMed/MEDLINE database search	Cardiovascular inflammation / circulating IL-37

10	[10]	34429116	Li L.	2021	PubMed/MEDLINE database search	Th1/Th2 regulation / endometriosis model
11	[11]	38583255	Shao	2024	Backward citation searching/reference-list checking	Transplantation / immune regulation
12	[12]	38159090	Qin	2024	Backward citation searching/reference-list checking	Immunotherapy / chronic allograft vasculopathy
13	[13]	39563375	Yazdani	2024	PubMed/MEDLINE database search	CNS autoimmunity / Treg and CD4+ responses
14	[14]	35741608	Li X.	2022	PubMed/MEDLINE database search	CNS diseases / IL-37 review
15	[15]	36040311	Lonnemann	2022	PubMed/MEDLINE database search	Alzheimer model / neuroinflammation
16	[16]	34702760	Zhou	2021	PubMed/MEDLINE database search	Atopic dermatitis / IL-37 in skin
17	[17]	40209639	Li Y.	2025	Backward citation searching/reference-list checking	IL-37 extracellular and intracellular signaling
18	[18]	40439750	Zhang J.	2025	Backward citation searching/reference-list checking	Microglia / LPS-induced neuroinflammation
19	[19]	36613827	Borgia	2022	PubMed/MEDLINE database search	IL-33/IL-37 axis / skin and allergic diseases
20	[20]	37589439	Wulamujiang	2023	Backward citation searching/reference-list checking	Psoriasis / IL-37 expression

21	[21]	39567940	Yu	2024	Backward citation searching/reference-list checking	Direct clinical psychiatric evidence / MDD and schizophrenia
22	[22]	38812484	Chen	2024	PubMed/MEDLINE database search	Chronic variable stress / depressive-like behavior
23	[23]	37206550	Xu J.	2023	PubMed/MEDLINE database search	Ferroptosis / diabetic atherosclerosis
24	[24]	39684587	Lyu	2024	PubMed/MEDLINE database search	Th17/Treg / arthritis model
25	[25]	33391457	Sánchez-Fernández	2021	Backward citation searching/reference-list checking	EAE / CNS autoimmunity
26	[31]	37690572	Su Z.	2023	Backward citation searching/reference-list checking	IL-37 / T-cell inhibition
27	[35]	34367169	Santarelli	2021	PubMed/MEDLINE database search	Circulating IL-37 reference range
28	[36]	40250016	Wang L.	2025	Backward citation searching/reference-list checking	Regulatory B cells / IL-37
29	[37]	38915806	Xu Y.	2024	PubMed/MEDLINE database search	IL-1 family cytokines / arthritis
30	[38]	40615556	Lee	2025	PubMed/MEDLINE database search	IL-37 / systemic lupus erythematosus
31	[39]	37834081	Tsuji	2023	PubMed/MEDLINE database search	IL-33 - IL-37 axis / atopic dermatitis and psoriasis

32	[40]	39126010	Rusiñol	2024	PubMed/MEDLINE database search	IL-18 and IL-37 / atopic dermatitis and psoriasis

This table lists the 32 sources of evidence included in the formal scoping review synthesis: 22 identified through the PubMed/MEDLINE database search and 10 identified through backward citation searching or reference-list checking. Eight contextual psychiatric publications that did not assess IL-37 were cited solely as background and were not included in the PRISMA screening pool; these publications are reported separately in Supplementary Table S3B. Methodological references concerning PRISMA 2020 and PRISMA-ScR were used only to describe the reporting standard and were not included in the substantive synthesis.
