# Supplementary material for: IL-37 and Neuroimmune Mechanisms Relevant to Depressive and Anxiety Disorders: A Scoping Review
Source: Int J Mol Sci. 2026 Jul 22;27(14):6496. doi: 10.3390/ijms27146496 (PMC13409887; doi:10.3390/ijms27146496)
Supplement: Supplementary file 1 [file ijms-27-06496-s001.zip › Supplementary_Table_S3A.pdf]

Supplementary Table S3A.
IL-37-related reports not present in the primary PubMed/MEDLINE exports and included in the formal scoping-review synthesis (n = 10).

This table lists 10 IL-37-related reports that were not present in the merged PubMed/MEDLINE exports reported in Supplementary Table S1A. Each report was reassessed against the same predefined eligibility criteria as the database-derived reports. The actual route and source of identification are reported for each publication. PubMed searches by title, author, and PMID were used solely to verify bibliographic details and were not treated as an independent identification method.

No.	Reference no. in manuscript	PMID	First author	Year	Identification method	Rationale for addition	Evidence category
1	[2]	34248996	Su	2021	Targeted PMID search / bibliography verification	Added because the publication provides a key review of IL-37 biology in human health and disease and was required to support the general mechanistic background of the review.	IL-37 biology / mechanistic background
2	[11]	38583255	Shao	2024	Targeted PMID search / bibliography verification	Added because the publication provides preclinical evidence concerning recombinant human IL-37 and immune regulation in transplantation, supporting the	IL-37 biology / immune regulation

						interpretation of IL-37-mediated immunoregulatory mechanisms.	
3	[12]	38159090	Qin	2024	Targeted PMID search / bibliography verification	Added because the publication provides evidence concerning IL-37 in immunotherapeutic mechanisms and chronic allograft vasculopathy, supporting the broader interpretation of IL-37-related immune regulation.	IL-37 biology / immune regulation
4	[17]	40209639	Li Y.	2025	Targeted PMID search / bibliography verification	Added because the publication describes extracellular and intracellular IL-37 signaling pathways, which were required for the mechanistic interpretation of IL-37 activity in the review.	IL-37 signaling / mechanistic background
5	[18]	40439750	Zhang J.	2025	Targeted PMID search / bibliography verification	Added because the publication provides preclinical evidence concerning IL-37, microglial phenotype	Preclinical neuroinflammation / microglia

						modulation, and LPS-induced neuroinflammation through the MyD88/NF-κB pathway.	
6	[20]	37589439	Wulamujiang	2023	Targeted PMID search / bibliography verification	Added because the publication provides dermatological evidence concerning IL-37 expression in psoriasis, supporting the peripheral inflammatory context and Figure 3.	Dermatology / peripheral inflammatory context
7	[21]	39567940	Yu	2024	Targeted PMID search / bibliography verification	Added because the publication provides the direct clinical psychiatric evidence in which IL-37 was included in the cytokine panel assessed in patients with major depressive disorder and schizophrenia.	Direct clinical psychiatric evidence
8	[25]	33391457	Sánchez-Fernández	2021	Targeted PMID search / bibliography verification	Added because the publication provides preclinical CNS autoimmunity evidence concerning IL-37 activity in experimental	CNS autoimmunity / indirect neuroimmune evidence

						autoimmune encephalomyelitis, relevant to indirect neuroimmune mechanisms.	
9	[31]	37690572	Su Z.	2023	Targeted PMID search / bibliography verification	Added because the publication provides mechanistic evidence concerning IL-37 upregulation and T-cell inhibition, supporting the interpretation of IL-37-related lymphocyte regulation.	IL-37 signaling / lymphocyte regulation
10	[36]	40250016	Wang L.	2025	Targeted PMID search / bibliography verification	Added because the publication provides evidence concerning IL-37-mediated regulatory B-cell activity and autoimmune suppression, supporting the interpretation of IL-37-related adaptive immune regulation.	IL-37 biology / regulatory B cells

These 10 reports were not present in the merged PubMed/MEDLINE exports. They were identified during the documented bibliography completeness audit and/or reference-list checking and were reassessed against the same predefined eligibility criteria as database-derived reports. PubMed searches by title, author, and PMID were used solely to verify bibliographic details and were not treated as an independent identification method. All 10 reports were included in the formal scoping-review synthesis and in the final PRISMA count of 32 sources of evidence.
