# Supplementary material for: IL-37 and Neuroimmune Mechanisms Relevant to Depressive and Anxiety Disorders: A Scoping Review
Source: Int J Mol Sci. 2026 Jul 22;27(14):6496. doi: 10.3390/ijms27146496 (PMC13409887; doi:10.3390/ijms27146496)
Supplement: Supplementary file 1 [file ijms-27-06496-s001.zip › Supplementary_Table_S3B.pdf]

Bdbievsfodbfpb

No.	Ref.	PMID	First author	year	Contextual topic	Role in the manuscript	IL-37 directly assessed	Included in PRISMA	Included in formal synthesis
1	[26]	37252156	Hassamal	2023	Chronic stress / neuroinflammation / depression	General background concerning the stress–inflammation–depression relationship and neuroinflammatory mechanism	---	---	---
2	[27]	38043258	Annam	2024	MDD / cytokine response to stress	General background concerning the stress–inflammation–depression relationship and neuroinflammatory mechanism	---	---	---
3	[28]	38176531	Chang	2024	Stress-induced depression / cytokines	Contextual review of inflammatory mechanisms linking chronic stress and	---	---	---

						depressive phenotypes			
4	[29]	33498653	Gałecka	2021	MDD / inflammatory and anti-inflammatory profiles	Contextual review of inflammatory mechanisms linking chronic stress and depressive phenotypes	---	---	---
5	[30]	35407663	Gałecka	2022	Depression / JAK–STAT pathway	Background concerning cytokine imbalance, Th17/Treg-related mechanisms, IL-35, and Foxp3 in depression	---	---	---
6	[33]	38308225	Liang	2024	MDD with/without anxiety / cytokine profile	Context concerning intracellular cytokine signaling and immune dysregulation in depressive disorders	---	---	---
7	[34]	35194013	Quagliato	2022	Panic disorder / cytokine profile	Epidemiological context concerning symptom-specific associations between inflammation, depression, and anxiety	---	---	---

8.	[32]	34135474	Milaneschi	2021	Inflammation / depression and anxiety	Clinical context concerning pro- and anti-inflammatory cytokine imbalance in anxiety disorders	---	---	---
